# Optimizing Care for Growth and Puberty in Duchenne Muscular Dystrophy: A Survey of Clinical Practice in the OPTIMIZE DMD Consortium

**DOI:** 10.1002/mus.70288

**Published:** 2026-05-24

**Authors:** Claire L. Wood, Funmbi Babalola, Robert W. Benjamin, Carol Lam, Laura McAdam, Stefan Nicolau, Anne Marie Sbrocchi, Mena Scavina, Julia C. Sorbara, Alexandra Ahmet, Susan Apkon, Sonum Bharill, Melissa Fiscaletti, Kathi Kinnett, Hugh J. McMillan, Nadia Merchant, Nat Nasomyont, Maria Fernanda Ochoa Molina, Nora E. Renthal, Pamela Smith, Prasanth N. Surampudi, Jaclyn G. Tamaroff, Cuixia Tian, Leanne M. Ward, David R. Weber, Sze Choong (Jarod) Wong, Janet L. Crane, Meilan M. Rutter, the OPTIMIZE DMD Consortium, Leanne M. Ward, Pat Furlong, David R. Weber, Sze Choong (Jarod) Wong, Hugh J. McMillan, Meilan M. Rutter, Susan Apkon, Roozbeh Pourziaei Manesh, Mei Wang, Anne Marie Sbrocchi, David R. Weber, Alexandra Ahmet, Aravindhan Veerapandiyan, Medical Sciences, Hugh J. McMillan, Sze Choong (Jarod) Wong, Kathryn A. Selby, Maria‐Elena Lautatzis, Rachel Schrader, Kathi Kinnett, Meilan M. Rutter, Claire L. Wood, Anne Marie Sbrocchi, Carol Lam, Funmbi Babalola, Janet L. Crane, Julia C. Sorbara, Laura McAdam, Robert Benjamin, Stefan Nicolau, Mena Scavina, Leanne M. Ward, Sze Choong (Jarod) Wong, Kim Phung, Nat Nasomyont, Anne Marie Sbrocchi, Cuixia Tian, David R. Weber, Janet L. Crane, Maria Fernanda Ochoa Molina, Meilan M. Rutter, Melissa Fiscaletti, Nadia Merchant, Paula R. Clemens, Rana Halloun, Shipra Bansal, Stefan Nicolau, Pat Furlong, Susan Apkon, Nora E. Renthal, Amanda M. Appel, Aravind Veerapandiyan, Medical Sciences, Claire L. Wood, Gabriela Vargas, Lindsey E. Bratland, Nat Nasomyont, Natalie Truba, Prasanth N. Surampudi, Janet Hoskin, Colin Werth, Patrick Moeschen, Nadia Merchant, Jaclyn Tamaroff, Christopher Lewis, Cuixia Tian, Diana X. Bharucha‐Goebel, Philip Zeitler, Laura McAdam, Maria‐Elena Lautatzis, Meilan M. Rutter, Ryan Farrell, Rainbow Babies, Rana Halloun, Sonum Bharill, Sze Choong (Jarod) Wong, Pat Furlong, Luca Bello, Chiara Panicucci, Clelia Cipolla, Danilo Fintini, Natascia Di Iorgi, Child Health, Sabrina Corbetta, Francesca Cumbo, Alberto Ferlin, Francesco Francini, Luisa De Sanctis, Tommaso Aversa, Gianluca Tornese, Burlo Garofolo, Eugenio Maria Mercuri, Elena Mularoni, Maria Grazia D'Angelo, Bosisio Parini, Chiara Arpaia, Silvia Carrara, Pieve Emanuele, Ilaria Zito, Fernanda de Angelis, Fabiana Boccia, Gaia Relucenti, Filippo Buccella

**Affiliations:** ^1^ Department of Paediatric Endocrinology Great North Children's Hospital Newcastle upon Tyne UK; ^2^ Translational and Clinical Research Institute Faculty of Medical Sciences, Newcastle University Newcastle upon Tyne UK; ^3^ Department of Pediatrics, Division of Endocrinology Western University, Children's Hospital, London Health Sciences Centre Ontario Canada; ^4^ Department of Pediatrics, Division of Endocrinology Duke University Medical Center Durham North Carolina USA; ^5^ Department of Pediatrics, University of Toronto, Canada; Division of Endocrinology Hospital for Sick Children Toronto Canada; ^6^ Bloorview Research Institute Holland Bloorview Kids Rehabilitation Hospital Toronto Canada; ^7^ Department of Paediatrics, Temerty Faculty of Medicine University of Toronto Toronto Canada; ^8^ Center for Gene Therapy, Nationwide Children's Hospital, Columbus, Ohio, USA; Department of Pediatrics The Ohio State University Columbus Ohio USA; ^9^ Division of Pediatrics and Metabolism Montreal Children's Hospital, McGill University Health Center Montreal Canada; ^10^ Parent Project Muscular Dystrophy Washington D.C USA; ^11^ Division of Endocrinology, Children's Hospital of Eastern Ontario (CHEO); Department of Pediatrics University of Ottawa Ottawa Canada; ^12^ Department of Physical Medicine and Rehabilitation Children's Hospital Colorado and University of Colorado School of Medicine Aurora Colorado USA; ^13^ Division of Endocrinology, Department of Pediatrics Johns Hopkins University School of Medicine Baltimore Maryland USA; ^14^ Department of Pediatrics Sainte Justine University Hospital, Université de Montréal Montreal Canada; ^15^ Division of Neurology, Children's Hospital of Eastern Ontario (CHEO); Department of Pediatrics University of Ottawa Ottawa Canada; ^16^ Division of Pediatric Endocrinology, Department of Pediatrics University of Texas Southwestern Medical Center Dallas Texas USA; ^17^ Division of Diabetes and Endocrinology, Cincinnati Children's Hospital Medical Center; College of Medicine University of Cincinnati Cincinnati Ohio USA; ^18^ Endocrinology Unit, Division of Pediatrics, School of Medicine Pontifical Catholic University of Chile Santiago CL Chile; ^19^ Department of Pediatrics, Division of Endocrinology Boston Children's Hospital and Harvard Medical School Boston Massachusetts USA; ^20^ Division of Endocrinology Phoenix Children's Hospital Phoenix Arizona USA; ^21^ Department of Internal Medicine, Division of Endocrinology University of California, Davis Medical Center Sacramento California USA; ^22^ Division of Endocrinology and Diabetes, Department of Pediatrics Vanderbilt University Medical Center Nashville Tennessee USA; ^23^ Division of Endocrinology and Diabetes, the Children's Hospital of Philadelphia; Department of Pediatrics University of Pennsylvania Perelman School of Medicine Philadelphia Pennsylvania USA; ^24^ Department of Pediatric Endocrinology, Royal Hospital for Children; Human Nutrition, School of Medicine, Dentistry and Nursing University of Glasgow Glasgow Scotland, UK; ^25^ Department of Pediatrics, Division of Endocrinology and Department of Orthopedic Surgery, Center for Musculoskeletal Research The Johns Hopkins University School of Medicine Baltimore Maryland USA

**Keywords:** Duchenne, endocrine, growth, hypogonadism, puberty

## Abstract

**Introduction/Aim:**

*Optimizing Management of Endocrine Complications in Duchenne Muscular Dystrophy* (OPTIMIZE DMD) is an international consortium of clinicians created to advance endocrine and bone clinical care in DMD. The aim of this study was to better understand current views and practices regarding investigation and management of growth and puberty concerns in individuals with DMD, relative to the 2018 Care Considerations and to inform updated guidance around endocrine care.

**Methods:**

A survey was created and sent to 47 OPTIMIZE DMD Consortium members and allied clinicians between September and November 2024. Areas surveyed included evaluation of growth, puberty, and arrested puberty/hypogonadism, and related management including use of vamorolone, growth hormone and testosterone.

**Results:**

Survey responses were received from 37 clinicians (79%). Most individuals were referred to endocrinology for growth and puberty concerns, with referral patterns contingent upon the endocrinology/multidisciplinary clinic model. Management discussions for growth concerns involved continued monitoring (95% of clinicians), glucocorticoid adjustment (54%) including vamorolone (35%), and growth hormone (41%). For pubertal delay, 88% of endocrinologists offered testosterone for pubertal induction, most commonly intramuscular 4‐weekly testosterone injections. Most endocrinologists (92%) offered testosterone supplementation for arrested puberty/hypogonadism, some (24%) on a case‐by‐case basis. Delivery routes were more varied, with subcutaneous injections prescribed by 52%.

**Discussion:**

Compared with the 2018 Care Considerations, monitoring practices for growth and puberty remain largely consistent, while variability persists in management approaches. These findings provide important insights to inform future guidance and identify priorities for further education and research in endocrine management for individuals with DMD.

**Abbreviations**
DMD: Duchenne muscular dystrophy
GH: Growth hormone
IM: Intramuscular
SC: Subcutaneous
UK: United Kingdom
US: United States

## 1 Introduction

Glucocorticoids are considered standard of care to slow disease progression in Duchenne muscular dystrophy (DMD). However, their use is associated with substantial endocrine side effects, including growth failure and pubertal delay [1].

*Optimizing Management of Bone Health and Endocrine Complications in DMD* (OPTIMIZE DMD) is an international consortium, founded in 2023 to advance the standard of care through education, advocacy, and research in endocrine and bone clinical care in DMD [2]. It comprises endocrinologists, neurologists, rehabilitation medicine clinicians, clinical psychologists, and patient advocates from the USA, Canada and the UK.

The objective of the study was to identify areas of consensus and variance amongst clinicians internationally and relative to the 2018 DMD Care Considerations [3, 4] (evidence‐ and expert opinion‐based guidance for DMD clinical care). The overarching goals were to determine current practices and inform future clinical guidance and research priorities in these domains.

## 2 Methods

A survey (supplemental document) was created and data analyzed by the OPTIMIZE DMD Growth and Puberty Working Group using Jisc v3, an online platform [5]. It was reviewed by The John Hopkins Medicine Institutional Review Board and determined exempt under US regulations. The survey was distributed electronically to the Consortium's members and allied Italian clinicians with DMD expertise between September and November 2024. The Italian clinicians were surveyed as they were participants of the Optimize DMD Rome 2025 meeting that was jointly organized by US and Italian patient advocacy organizations. Areas surveyed included monitoring, referrals, investigation and management of growth and puberty concerns in younger individuals and arrested puberty/hypogonadism in older teenagers and young adults. Management strategies discussed included the use of vamorolone, growth hormone and testosterone. For referrals and monitoring, all responses were analyzed, whereas for endocrine‐focused investigations and management following endocrinology referral, endocrinologists' responses only were analyzed.

## 3 Results

### 3.1 Demographics

Survey responses were received from 37/47 clinicians (79%) with expertise in managing DMD (Table 1). All non‐responders were sent three reminder emails.

**TABLE 1:** Demographics of survey respondents.

Characteristic	Survey response rate, *n* (%)
Overall	37/47 (79)
OPTIMIZE DMD members	28/35 (80)
Italian clinicians	9/12 (75)
Country of practice	
USA	17 (46)
Canada	9 (24)
Italy	9 (24)
UK	2 (5)
Clinical specialty	
Pediatric endocrinologist	25 (68)
Neurologist/neuromuscular specialist	10 (27)
Pediatrician (DMD expertise)	1 (3)
Clinical psychologist (DMD expertise)	1 (3)
DMD patient caseload	
< 10 patients	3 (8)
51–200 patients	22 (60)
> 200 patients	5 (14)
Age groups cared for	
Children with DMD	36 (97)
Adults only	1 (3)
Continue care to ages 19–22 years	25 (68)
Continue care beyond 22 years	16 (43)
Multidisciplinary DMD clinic (v separate endocrine clinic)	
USA	10 (63)
Canada	2 (22)
Italy	3 (33)
UK	1 (50)

Abbreviation: DMD: Duchenne muscular dystrophy.

### 3.2 Growth

Eight respondents (22%) reported that an endocrinologist routinely saw all patients in their multidisciplinary clinic and thus no specific referral for investigation of growth concerns was needed. Twenty‐seven of the remaining 29 respondents reported referral to endocrinology for growth concerns (from patient, family, or physician).

**FIGURE 1:**
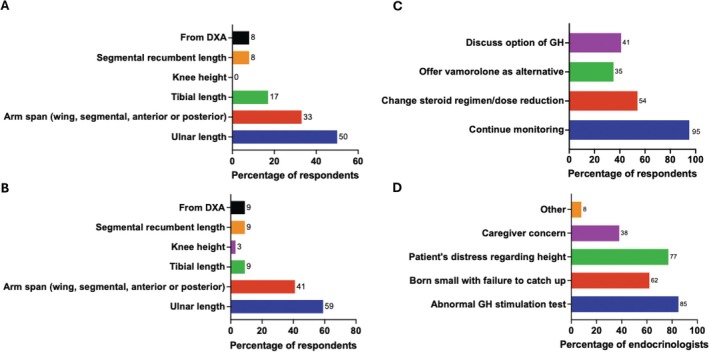
(1A) Methods for measurement of non‐standing height in ambulant individuals performed by 12/37 clinicians; (1B) Methods for measurement of non‐standing height in non‐ambulant individuals performed by 32/37 clinicians; (1C) Options discussed with patient when there are growth concerns; (1D) Reasons for treatment with growth hormone (GH), responses from endocrinologists only.

All clinicians monitored growth using standing height measurements in ambulant individuals, usually every 6–12 months. Thirty‐two of 37 (86%) routinely measured surrogates of height in non‐ambulant individuals but only 12/37 (32%) also did this in ambulant individuals. These surrogate methods were highly variable (Figure 1A,B).

Bone age x‐rays constituted part of the growth assessment by 23/25 (92%) endocrinologists. On referral, 23/25 (92%) endocrinologists initiated standard biochemical testing to investigate growth (including renal and thyroid function, growth hormone (GH) indices, erythrocyte sedimentation rate, celiac screen and complete blood count). Growth hormone provocation testing was considered by 56% of endocrinologists on a case‐by‐case basis (if suspicion of GH deficiency and/or if considering GH treatment), 28% performed it routinely for impaired growth, and 16% did not offer it.

Regarding management discussions, 95% of clinicians discussed monitoring without intervention, 54% discussed adjusting the current glucocorticoid regimen, 35% discussed changing to vamorolone, and 41% discussed GH (Figure 1C). Use of GH was stratified by country from two‐thirds of US and Italian endocrinologists reporting its use, to minimal use by Canadian and UK endocrinologists, and was usually on a case‐by‐case basis (86%). Reasons for treatment with GH are highlighted in Figure 1D.

### 3.3 Delayed Puberty

All centres referred to endocrinology for delayed puberty, with 10/37 (27%) already seeing the endocrinologist within the multidisciplinary DMD clinic and the remaining 27 having patients referred specifically for concerns regarding pubertal progression. The age at referral varied from < 12 (19%), 12–14 (73%), and > 14 years (8%); 35/37 clinicians referred by 14 years of age. Most respondents (91%) said only endocrinology performed pubertal staging by clinical examination and thus age of first pubertal examination correlated with age at referral.

Clinical tests by endocrinology to assess delayed puberty included serum testosterone (22/25, 88%), gonadotropins—luteinizing hormone and follicle stimulating hormone (20/25, 80%), Sertoli cell markers—inhibin B and anti‐Mullerian hormone (4/25, 16%), and bone age x‐ray (16/25, 64%). The most common age for first testosterone measurement was 12 years (9/25, 41%) but was not performed by 3 (14%) endocrinologists until 14 years of age.

All but one endocrinologist reported routinely engaging in shared decision‐making discussions regarding options of continued monitoring versus testosterone therapy. One respondent, a psychologist, articulated the importance of individualized care: “It is important to consider both temperament and emotional impact of each choice […] relative to the IQ and neurodevelopmental characteristics of each individual patient.”

**FIGURE 2:**
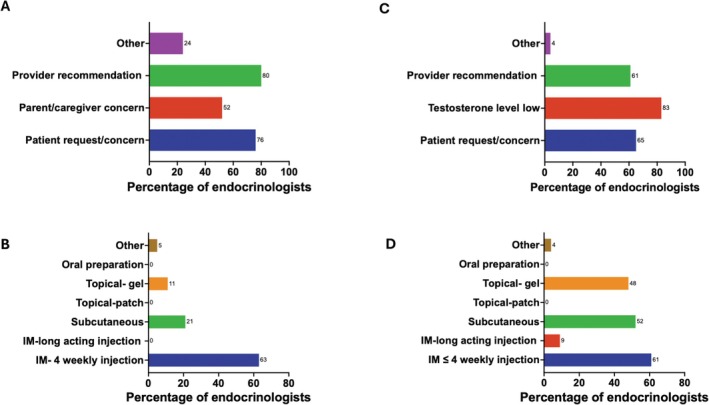
(2A) Reasons for offering testosterone supplementation for pubertal induction in younger adolescents; (2B) Routes of testosterone supplementation used for pubertal induction in younger adolescents; IM: Intramuscular; (2C) Reasons for offering testosterone supplementation for arrested puberty or hypogonadism in older teenagers/young adults; (2D) Route of testosterone supplementation in older teenagers/young adults; IM: Intramuscular.

Sixty‐eight % (17/25) of endocrinologists routinely offered testosterone treatment for pubertal induction, whilst 5 (20%) offered it on an individual basis, and 3 (12%) did not routinely offer it. Bone health was cited as a reason to consider treatment in addition to delayed puberty. The age at which testosterone supplementation was offered was variable but typically occurred at 14 years and was based on a combination of provider recommendation and patient or parent concern (Figure 2A). Intramuscular 4‐weekly injections were the most common route of testosterone replacement for pubertal induction (Figure 2B).

### 3.4 Arrested Puberty/Hypogonadism

Testosterone (23/25, 92%) and gonadotrophin levels (18/25, 72%) were routinely measured by the surveyed endocrinologists in older teenagers/young adults who had spontaneous puberty onset or been previously treated with testosterone to induce puberty. Only 4 (16%) endocrinologists routinely measured Sertoli cell markers. Testosterone supplementation was offered by 23/25 endocrinologists (92%) for arrested puberty/hypogonadism, with 6 (24%) doing so on a case‐by‐case basis. Reasons for offering testosterone supplementation to this older population included patient request or concern, low serum testosterone concentrations and/or provider recommendation (Figure 2C).

Routes of testosterone administration offered were more varied for older individuals with arrested puberty/hypogonadism (Figure 2D). Route varied by country, with subcutaneous injections widely used in the US and Canada (13/17, 76%) but not in the UK or Italy. Longer‐acting (3‐monthly) intramuscular testosterone injections were only used in the UK. Topical gel use was predominant in Italy.

## 4 Discussion

Compared with the 2018 Care Considerations, monitoring practices for growth and puberty remained largely consistent, while variability persisted in management approaches. Recurring themes in this cross‐sectional survey included proactive investigation and management, particularly of delayed puberty, and individualized patient‐centred care involving shared, interdisciplinary decision‐making.

There was variability in the approach to counseling and management of growth concerns. For example, use of GH varied by country, which may be due to differences in accessibility/licensing indications, insurance coverage, as well as lack of long‐term controlled studies on safety and efficacy of GH in this population [6, 7, 8]. In accordance with the 2018 Care Considerations [3, 4], surveyed endocrinologists did not prescribe GH therapy as routine standard care, but on a case‐by‐case basis, underscoring the understandable concern from patients and families about impaired growth.

The use of vamorolone, a new dissociative glucocorticoid, may become more widespread due to its growth‐sparing effects seen over 3 years in clinical studies [9, 10, 11] and deserves consideration in future guidance. Discussions regarding its use for growth failure were already taking place by some of those surveyed, but further data are required.

There was consensus around pubertal induction by age 14 years, as recommended by the 2018 Care Considerations [3], with variance in the age of initial assessment that could inform updated guidance. Beyond the practice of pubertal induction, surveillance of androgen production and testosterone supplementation in young adults with DMD is not mentioned in the 2018 Care Considerations [3]. This survey demonstrates that this is now common practice amongst experts in the field, and there is increasing evidence around the systemic benefits of testosterone, emphasizing an area for inclusion and further exploration in future guidance [8, 12, 13, 14, 15, 16].

A limitation of this survey was the relatively small number of clinicians (mainly from North America) surveyed, which could affect generalizability [17, 18, 19, 20] and skew results. We also acknowledge that expert practice may not be representative of wider practice in the areas surveyed and may depend on knowledge and resources available in different centres.

## 5 Conclusions

There are areas of consensus in the management of growth and puberty/hypogonadism in individuals with DMD, which should inform updated guidance around endocrine care. Variability in management strategies highlight areas for research, including the long‐term impact of GH therapy, the optimal (possibly earlier) timing for pubertal induction given the likelihood of pubertal delay in those receiving glucocorticoids, and surveillance and management of post‐pubertal hypogonadism. A wider international survey of more clinicians involved in care and not just ‘experts in the field’ would help to further delineate consensus and variation in practice and inform optimal care.

## Author Contributions

**Laura McAdam:** writing – review and editing. **Anne Marie Sbrocchi:** writing – review and editing. **Stefan Nicolau:** writing – review and editing. **Julia C. Sorbara:** writing – review and editing. **Susan Apkon:** writing – review and editing. **Melissa Fiscaletti:** writing – review and editing. **Funmbi Babalola:** writing – review and editing. **Robert W. Benjamin:** writing – review and editing. **Alexandra Ahmet:** writing – review and editing. **Carol Lam:** writing – review and editing. **Claire L. Wood:** conceptualization, writing – original draft, data curation, formal analysis, writing – review and editing. **Mena Scavina:** writing – review and editing. **Sonum Bharill:** writing – review and editing. **Nora E. Renthal:** writing – review and editing. **Maria Fernanda Ochoa Molina:** writing – review and editing. **Nat Nasomyont:** writing – review and editing. **Kathi Kinnett:** writing – review and editing. **Nadia Merchant:** writing – review and editing. **Pamela Smith:** writing – review and editing. **Hugh J. McMillan:** writing – review and editing. **Cuixia Tian:** writing – review and editing. **Sze Choong (Jarod) Wong:** writing – review and editing. **Leanne M. Ward:** writing – review and editing, supervision. **Prasanth N. Surampudi:** writing – review and editing. **Meilan M. Rutter:** writing – review and editing, conceptualization, data curation, formal analysis. **David R. Weber:** writing – review and editing. **Janet L. Crane:** writing – review and editing. **Jaclyn G. Tamaroff:** writing – review and editing.

## Funding

The authors received no financial support for the research, authorship, or publication of this article. Dr. Leanne M. Ward is supported by a Senior Research Chair in Pediatric Genetic and Metabolic Bone Disorders from the University of Ottawa and the Children's Hospital of Eastern Ontario Research Institute.

## Ethics Statement

We confirm that we have read the Journal's position on issues involved in ethical publication and affirm that this report is consistent with those guidelines.

## Conflicts of Interest

C.W. served as a Consultant for Roche. L.M. was a co‐investigator for the vamorolone clinical trial. M.S. served as a consultant for Pfizer in 2023 and has been an advisor/consultant for PPMD. A.A. has received honoraria for consultancy work from Reveragen, with funds to Dr. Ahmet's Institution. M.F. has received grant support from the University of Montreal, Azrieli Research Centre of CHU Sainte Justine, and Muscular Dystrophy Canada. H.J.M. has consulted for Roche, Kye Pharma, Regenxbio, and Solid Biosciences. He has been a site principal investigator for clinical trials sponsored by Roche, Sarepta, Italfarmaco, Dyne Therapeutics, PepGen, Regenxbio, Pfizer, and Reveragen. N.M. has been on the advisory board for BioMarin, Catalyst, Pfizer, BridgeBio, Ascendis, Kyowa Kirin, and Alexion. P.S. has been a consultant and received speaker honoraria from Biomarin. P.N.S. has participated in advisory board meetings for Santhera Pharmaceutical (2025) and Catalyst (2024). J.T. was a Consultant for Catalyst Pharmaceuticals (Pediatric Endocrine Advisory Board May 2024). C.T. served as a Consultant for Biogen, Catalyst, Entrata, Itf, Pfizer, Sarepta and received research funding from AveXis/Novartis, Biohaven, Capricor, Genentech/Roche, Roche, PTC and Sarepta. L.M.W. has been a consultant to Amgen, Ultragenyx, Kyowa Kirin, Roche, Angitia, Santhera, Catalyst, Biomarin, and Ipsen, with funds to Dr. Ward's institution, and has participated in clinical trials with Alexion, QED, Ultragenyx, Edgewise, ReveraGen, Ascendis, Roche, and Catalyst, with funds to Dr. Ward's Institution. D.R.W. has been a consultant for Catalyst, Inozyme, and Santhera. S.C.W. has been a consultant for Santhera, Roche, and Novartis, and has received speaker honoraria from Nutricia, Sandoz, Novo Nordisk, and Roche. J.C. served on an advisory board for Syneous Health. MR served as a Consultant for Catalyst Pharmaceuticals Inc. F.B., R.B., C.L., S.N., A.M.S., J.S., S.A., S.B., K.K., N.N., M.F.O.M., and N.E.R. have no COI related to this paper to disclose.

## Supporting information


**Data S1:** Optimize DMD growth and puberty survey.

## Data Availability

The data that support the findings of this study are available from the corresponding author upon reasonable request.

## References

[1] L. M. Ward and D. R. Weber , “Growth, Pubertal Development, and Skeletal Health in Boys With Duchenne Muscular Dystrophy,” Current Opinion in Endocrinology, Diabetes, and Obesity 26, no. 1 (2019): 39–48. 30507696 10.1097/MED.0000000000000456 PMC6402320

[2] L. M. Ward , D. R. Weber , S. C. Wong , et al., “A Parent Project Muscular Dystrophy‐Sponsored International Workshop Report on Endocrine and Bone Issues in Patients With Duchenne Muscular Dystrophy: An Ever‐Changing Landscape,” J Neuromuscul Dis 12, no. 1 (2025): 303370. 10.1177/22143602241303370 PMC11949309 39973454

[3] D. J. Birnkrant , K. Bushby , C. M. Bann , et al., “Diagnosis and Management of Duchenne Muscular Dystrophy, Part 1: Diagnosis, and Neuromuscular, Rehabilitation, Endocrine, and Gastrointestinal and Nutritional Management,” Lancet Neurology 17, no. 3 (2018): 251–267. 29395989 10.1016/S1474-4422(18)30024-3 PMC5869704

[4] D. R. Weber , S. Hadjiyannakis , H. J. McMillan , G. Noritz , and L. M. Ward , “Obesity and Endocrine Management of the Patient With Duchenne Muscular Dystrophy,” Pediatrics 142, no. Suppl 2 (2018): S43–S52. 30275248 10.1542/peds.2018-0333F PMC6460463

[5] Jisc . Online surveys (2025).

[6] M. M. Rutter , J. Collins , S. R. Rose , et al., “Growth Hormone Treatment in Boys With Duchenne Muscular Dystrophy and Glucocorticoid‐Induced Growth Failure,” Neuromuscular Disorders 22, no. 12 (2012): 1046–1056. 22967789 10.1016/j.nmd.2012.07.009

[7] M. M. Rutter , B. L. Wong , J. J. Collins , et al., “Recombinant Human Insulin‐Like Growth Factor‐1 Therapy for 6 Months Improves Growth but Not Motor Function in Boys With Duchenne Muscular Dystrophy,” Muscle & Nerve 61, no. 5 (2020): 623–631. 32108355 10.1002/mus.26846

[8] E. Loscalzo , J. See , S. Bharill , et al., “Growth Hormone and Testosterone Delay Vertebral Fractures in Boys With Muscular Dystrophy on Chronic Glucocorticoids,” Osteoporosis International 35, no. 2 (2024): 327–338. 37872346 10.1007/s00198-023-06951-z PMC10837224

[9] M. Guglieri , P. R. Clemens , S. J. Perlman , et al., “Efficacy and Safety of Vamorolone vs Placebo and Prednisone Among Boys With Duchenne Muscular Dystrophy: A Randomized Clinical Trial,” JAMA Neurology 79 (2022): 1005–1014. 36036925 10.1001/jamaneurol.2022.2480 PMC9425287

[10] J. K. Mah , P. R. Clemens , M. Guglieri , et al., “Efficacy and Safety of Vamorolone in Duchenne Muscular Dystrophy: A 30‐Month Nonrandomized Controlled Open‐Label Extension Trial,” JAMA Network Open 5, no. 1 (2022): e2144178. 35076703 10.1001/jamanetworkopen.2021.44178 PMC8790668

[11] U. J. Dang , J. M. Damsker , M. Guglieri , et al., “Efficacy and Safety of Vamorolone Over 48 Weeks in Boys With Duchenne Muscular Dystrophy: A Randomized Controlled Trial,” Neurology 102, no. 5 (2024): e208112. 38335499 10.1212/WNL.0000000000208112 PMC11067696

[12] C. L. Wood , K. G. Hollingsworth , E. Bokaie , et al., “Is Ongoing Testosterone Required After Pubertal Induction in Duchenne Muscular Dystrophy?,” Endocr Connect 12, no. 12 (2023). 10.1530/EC-23-0245 PMC10620460 37768006

[13] C. L. Wood , J. Page , J. Foggin , M. Guglieri , V. Straub , and T. D. Cheetham , “The Impact of Testosterone Therapy on Quality of Life in Adolescents With Duchenne Muscular Dystrophy,” Neuromuscular Disorders 31, no. 12 (2021): 1259–1265. 34702655 10.1016/j.nmd.2021.09.007 PMC8721209

[14] C. L. Wood , K. G. Hollingsworth , E. Hughes , et al., “Pubertal Induction in Adolescents With DMD Is Associated With High Satisfaction, Gonadotropin Release and Increased Muscle Contractile Surface Area,” European Journal of Endocrinology 184, no. 1 (2021): 67–79. 33112266 10.1530/EJE-20-0709

[15] S. L.‐K. Lee , A. Lim , C. Munns , P. J. Simm , and M. Zacharin , “Effect of Testosterone Treatment for Delayed Puberty in Duchenne Muscular Dystrophy. *Horm* Res,” Paediatr 93, no. 2 (2020): 108–118. 10.1159/000508290 32610327

[16] G. Sodero , C. Cipolla , D. Rigante , F. Arzilli , and E. M. Mercuri , “Pubertal Induction Therapy in Pediatric Patients With Duchenne Muscular Dystrophy,” Journal of Pediatric Endocrinology & Metabolism 38 (2025): 781–787. 40068954 10.1515/jpem-2025-0061

[17] A. C. McPherson , R. Amin , L. McAdam , D. Kalnins , and T. Lui , “Growth Assessment and Weight Management in Paediatric Neuromuscular Clinics: A Cross‐Sectional Survey Across Canada,” Disability and Rehabilitation 43, no. 21 (2021): 3015–3020. 32058820 10.1080/09638288.2020.1725155

[18] D. Galetaki , V. Szymczuk , M. Shi , and N. Merchant , “Is Endocrine Surveillance Important in the Care of Duchenne Muscular Dystrophy? Results From a National Survey to Patients and Families on Endocrine Complications,” eNeurologicalSci 36 (2024): 100513. 38989274 10.1016/j.ensci.2024.100513 PMC11231648

[19] A. Henderson , G. Harley , I. Horrocks , et al., “Endocrine and Bone Monitoring in Boys With Duchenne Muscular Dystrophy; Do We Adhere to the Standards of Care?,” J Neuromuscul Dis 10, no. 6 (2023): 1143–1144. 37927273 10.3233/JND-230144 PMC10657663

[20] S. McCarrison , S. Abdelrahman , J. H. Davies , et al., “The Use of Bisphosphonate and Testosterone in Young People With Duchenne Muscular Dystrophy: An International Clinician Survey,” Journal of Pediatric Endocrinology & Metabolism 38 (2025): 807–814. 40270468 10.1515/jpem-2025-0039

