# Supplementary material for: Optimizing Care for Growth and Puberty in Duchenne Muscular Dystrophy: A Survey of Clinical Practice in the OPTIMIZE DMD Consortium
Source: Muscle Nerve. 2026 May 24;74(2):433–9. doi: 10.1002/mus.70288 (PMC13332569; doi:10.1002/mus.70288)
Supplement: Supplementary file 1 — Data S1: Optimize DMD growth and puberty survey. [file MUS-74-433-s001.pdf]

Optimize DMD growth and puberty survey

1. Please tell us your Name, specialty / role, and Institution *

2. Are patients referred to an Endocrinologist in your centre for any endocrine concerns (eg. puberty, weight, osteoporosis)? *

- ◯ Yes
- ◯ No
- ◯ On a case-by-case basis

If you answered 'yes' or 'on a case-by-case basis' to Q2, please proceed to Q3. If you answered 'no' please proceed to Q4

3. If yes, where do you see these patients?

- ◯ Dedicated endocrinologist in multidisciplinary clinic
- ◯ Dedicated endocrinologist in own endocrine clinic
- ◯ Any endocrinologist in own endocrine clinic

4. If you answered 'no' in Q2, would you be willing to change your practice to do this?

- ◯ Yes
- ◯ No
- ◯ Not sure

5. Are patients in your centre referred to endocrinology for growth concerns?

- ◯ Yes, please go to Q6
- ◯ No, please go to Q7
- ◯ Referred on a case-by-case basis, please go to Q6

◯ No referral needed- endocrinologist sees most/all patients in multidisciplinary clinic, , please go to Q7

6. If you answered 'yes' in Q5, what would be the reason for referral? (Please select as many as applicable)

☐ Patient concern
☐ Parent concern
☐ Physician concern
☐ All patients with growth deceleration or short stature referred routinely

7. If you answered 'no' in Q5 would you be willing to change your practice to do this?

◯ Yes
◯ No
◯ Not sure

8. Are patients in your centre referred to endocrinology for delayed puberty?

◯ Yes, please go to Q9
◯ No, please go to Q11
◯ Referred on a case-by-case basis, please go to Q9
◯ No referral needed- endocrinologist sees most/all patients in multidisciplinary clinic, please go to Q11
◯ Not usually referred for puberty – typically referred for other endocrine issue e.g. osteoporosis and puberty is addressed concurrently, please go to Q11

9. If you answered 'yes' in Q8, at what age are patients usually referred?

◯ 11 years
◯ 12 years
◯ 13 years
◯ 14 years
◯ Older than 14 years

10. If you answered 'yes' in Q8, what would be the reason for referral?

- ◯ Absent puberty on pubertal exam
- ◯ All patients by age of referral (no puberty exam)
- ◯ Other- please specifiy below

11. Is linear growth routinely measured in ambulatory patients in your centre?

- Yes
- No

If 'yes' please proceed to Q12, if no, please proceed to Q13

12. If yes, how frequently is this measured?

- 6-monthly
- 12-monthly
- As needed

13. If you do not routinely measure linear growth, would you be willing to change your practice to do it?

- Yes
- No
- Not sure

14. Is NON-standing height routinely measured in ambulatory patients?

- Yes
- No

If 'yes' please proceed to Q15, if no, please proceed to Q17

15. If yes, how frequently is this measured?

- 6-monthly
- 12-monthly

- ◯ As needed

16. If you measure NON-standing height in ambulatory patients, what method do you use?

- ◯ ulnar length
- ◯ arm span (wing, segmental, anterior or posterior)
- ◯ tibial length
- ◯ knee height
- ◯ segmental recumbent length
- ◯ from DXA

17. If you do not routinely measure NON-standing height in ambulatory patients, would you be willing to change your practice to do it?

- ◯ Yes
- ◯ No
- ◯ Not sure

18. Is NON-standing height routinely measured in non-ambulatory patients?

- ◯ Yes
- ◯ No

If 'yes' please proceed to Q19, if no, please proceed to Q21

19. If yes, how frequently is this measured?

- ◯ 6-monthly
- ◯ 12-monthly
- ◯ As needed

20. If you measure NON-standing height in non-ambulatory patients, what method do you use?

- ulnar length
- arm span (wing, segmental, anterior or posterior)
- tibial length
- knee height
- segmental recumbent length
- from DXA

21. If you do not routinely measure NON-standing height in non-ambulatory patients, would you be willing to change your practice to do it?

- Yes
- No
- Not sure

22. Do you use a bone age x-ray as part of your growth investigation?

- Yes
- No

If 'yes' please proceed to Q23, if no, please proceed to Q24

23. If yes, how frequently is this measured?

- Initial evaluation only
- 12-monthly
- Initial, then as needed thereafter

24. If you do not routinely measure bone age by xray, would you be willing to change your practice to do it?

- Yes
- No

◯ Not sure

25. Do you use any of the following biochemical tests to investigate growth? (Please tick all that apply)

◯ IGF-1 and/or IGFBP3
◯ Thyroid function tests
◯ Chronic disease screen (e.g. CBC, ESR, Celiac screen, renal function)

If 'yes' please proceed to Q26, if no, please proceed to Q27

26. If yes, how frequently is this measured?

◯ Initial evaluation only
◯ 12-monthly
◯ Initial, then as needed thereafter

27. If you do not routinely use biochemical tests, would you be willing to change your practice to do it?

◯ Yes
◯ No
◯ Not sure

28. Do you use growth hormone provocation testing to investigate growth failure? (e.g. arginine, glucagon, cllonidine, L-dopa tests)

◯ Yes
◯ No
◯ On a case-by-case basis

29. If you answered 'on a case-by-case basis' what would prompt provocation testing?

30. Do you discuss any of the following as options regarding growth concerns? Please tick all that apply

- Continue monitoring
- Change steroid regimen/dose reduction
- Offer vamorolone as alternative
- Discuss option of growth hormone

31. Do you routinely offer growth hormone in the setting of short stature/impaired growth in DMD?

- Yes
- No
- We consider it on a case-by-case basis

32. If you answered yes to Q31, what would be valid reasons for treatment with GH? (Please tick all that apply)

- Abnormal GH stimulation test
- Born small with failure to catch up
- Patient's psychological distress regarding height
- Caregiver concern
- Other

33. If you answered 'other' to Q32, please specify..

34. Is clinical examination of pubertal stage using Tanner staging and testicular volumes routinely done in your centre?

- ◯ Yes
- ◯ No

35. If you answered 'yes' to Q34, is this done before referral to endocrinology or after? If you answered 'no' please go to Q39

- ◯ Before referral to endocrinology
- ◯ Yes, but only after referral to endocrinology

36. Please detail who does the clinical examination if this occurs before referral..

37. At what age does clinical examination of pubertal stage start?

- ◯ 9 years
- ◯ 10 years
- ◯ 11 years
- ◯ 12 years
- ◯ 13 years
- ◯ 14 years or older

38. How often is clinical examination of pubertal staging carried out?

- 6 monthly
- 12 monthly
- As needed

39. If you don't routinely do clinical examination of pubertal staging would you be willing to change your practice to do it?

- Yes
- No

40. Do you routinely perform a bone age x-ray as part of your investigation of delayed puberty? If no, please go to Q42 *

◯ Yes
◯ No

41. If you answered 'yes' to Q 40 how often do you do a bone age x-ray?

◯ At initial evaluation only
◯ 12 monthly
◯ As needed after initial evaluation

42. If you do not routinely measure bone age, would you be willing to change your practice to do it?

◯ Yes
◯ No
◯ Not sure

43. Do you routinely measure gonadotrophins (LH and FSH) as part of your investigation of delayed puberty? If no, please go to Q46 *

◯ Yes
◯ No

44. If you answered 'yes' to Q 43 at what age do you first measure gonadotrophins?

◯ 9 years
◯ 10 years
◯ 11 years
◯ 12 years
◯ 13 years

- 14 years or older

45. If you answered 'yes' to Q 43 how often do you measure gonadotrophins?

- 6 monthly
- 12 monthly
- As needed

46. Do you routinely measure testosterone as part of your investigation of delayed puberty? If no, please go to Q50 *

- Yes
- No

47. If you answered 'yes' to Q 46 at what age do you first measure testosterone levels?

- 9 years
- 10 years
- 11 years
- 12 years
- 13 years
- 14 years or older

48. If you answered 'yes' to Q 46 how often do you measure testosterone levels?

- 6 monthly
- 12 monthly
- As needed

49. If you do not routinely measure testosterone levels, would you be willing to change your practice to do it?

- Yes
- No

- Not sure

50. Do you routinely measure Sertoli cell markers (Inhibin B/AMH) as part of your investigation of delayed puberty? If no, please go to Q54 *

- Yes
- No

51. If you answered 'yes' to Q 50 at what age do you first measure Sertoli cell markers?

- 9 years
- 10 years
- 11 years
- 12 years
- 13 years
- 14 years or older

52. If you answered 'yes' to Q 50 how often do you measure Sertoli cell markers?

- 6 monthly
- 12 monthly
- As needed

53. If you do not routinely measure Sertoli cell markers, would you be willing to change your practice to do it?

- Yes
- No

◯ Not sure

54. Do you counsel or engage in shared decision-making regarding options for delayed puberty (in younger adolescents)?

Options include:

1) continued monitoring

2) testosterone therapy (pros/cons) *

◯ Yes
◯ No

55. If you answered 'yes' to Q54, who discusses this and how frequently?

56. Do you measure testosterone levels in older teens/young adults who may have had spontaneous puberty onset or been previously treated with testosterone to induce puberty?

- ◯ Yes
- ◯ No

57. If you answered 'yes' to Q57, how often do you measure testosterone levels?

- ◯ 6 monthly
- ◯ 12 monthly
- ◯ As needed

58. Do you counsel or engage in shared decision-making regarding options for arrested puberty or hypogonadism (in older teens/ young adults who may have had spontaneous puberty onset or been previously treated with testosterone to induce puberty)?

Options include:

1) continued monitoring

2) testosterone therapy (pros/cons)

- ◯ Yes
- ◯ No

59. If you answered 'yes' to Q59, who discusses this and how frequently?

60. Do you offer testosterone supplementation for pubertal induction in younger adolescents

- ◯ Yes
- ◯ No
- ◯ On an individualised basis

61. If you answered 'yes' to Q 61, what is the minimum age at which you would offer testosterone supplementation?

- ◯ 11 years
- ◯ 12 years
- ◯ 13 years
- ◯ 14 years
- ◯ 15 years or older

62. If you answered 'yes' to Q 61, what are the reasons for offering testosterone?

- ☐ Patient request/concern
- ☐ Parent/caregiver concern
- ☐ Provider recommendation
- ☐ Other

63. If you answered 'no' to Q61, what are your reasons for not offering testosterone supplementation?

64. Do you offer testosterone supplementation for arrested puberty or hypogonadism (in older teens/young adults who may have had spontaneous puberty onset or been previously treated with testosterone to induce puberty

- ◯ Yes
- ◯ No
- ◯ On an individual basis

65. If you answered 'yes' to Q 65, what are the reasons for offering testosterone?

- ☐ Patient request/concern
- ☐ Testosterone level low
- ☐ Provider recommendation
- ☐ Other

66. If you answered 'no' to Q65, what are your reasons for not offering testosterone supplementation?
